# Liver Resection in Hepatitis B-Related Hepatocellular Carcinoma: Clinical Outcomes and Safety in Overweight and Obese Patients

**DOI:** 10.1371/journal.pone.0099281

**Published:** 2014-06-10

**Authors:** Haiqing Wang, Jian Yang, Xiaowu Zhang, Lunan Yan, Jiayin Yang

**Affiliations:** Department of Liver Surgery, West China Hospital of Sichuan University, Chengdu, China; Xiangya Hospital of Central South University, China

## Abstract

**Objective and Background:**

Although many studies on evaluating the safety of liver resection in obese patients have been conducted, the results remain contradictory. The aim of our study was to investigate the safety of overweight and obese patients undergoing liver resection for hepatitis B-related hepatocellular carcinoma in a large sample.

**Methods:**

In a retrospective cohort with 1543 hepatitis B-related hepatocellular carcinoma patients, the subjects were stratified into four groups according to their body mass index(BMI): obesity(BMI≥28), overweight(BMI:24.0–27.9), normal weight(BMI:18.5–23.9) and underweight(BMI<18.5). The Dindo–Clavien classification system was used for grading complications. Clinical characteristics and operative outcomes were compared among the four groups. Risk factors for postoperative complications were evaluated by multivariate analysis.

**Results:**

According to the category criteria of the Working Group on Obesity in China (WGOC) criteria, 73(4.7%) obese, 412(26.7%) overweight, 982(63.6%) normal weight and 76(4.9%) underweight patients were included in our cohort. Overweight and obese patients had more preoperative comorbidities such as hypertension(P<0.001). Mortality, total complications and complications classified by Clavien system were similar among the four groups except that the underweight patients had fewer total complications. However, postoperative wound complication was more common in overweight and obese patients(6.3% vs 2.5%,P<0.001,11.0% vs 2.5%,P = 0.001). Multivariate analysis revealed that BMI was not an independently significant factor for postoperative complications.

**Conclusions:**

Liver resection for obese and overweight patients is safe and BMI itself is not a risk factor for mortality and morbidity.

## Introduction

Liver resection remains one of the most common and effective treatments for hepatocellular carcinoma(HCC) and has become a widely accepted therapy method[1]. With the refinement of surgical techniques and perioperative management in liver surgery over the past several decades, outcomes after liver resection have improved substantially in recent years. However, the surgeons are facing with new challenge deriving from the longer and more difficult surgeries for obese and overweight patients[2]. With the prevalence of obesity continuously increasing worldwide, obesity and overweight are an increasing public health problem. In the United States[3], 66.2% of adults were obese or overweight in 2003–2004 year. Although there is a much lower prevalence of obesity and overweight in China than in other parts of the world, the condition has increased dramatically during recent decades[4], [5]. Obesity in Chinese adults, according to criteria from the Working Group on Obesity in China (WGOC) criteria, increased during the period from 1993 to 2009, from 2.9% to 8.7%, and the prevalence of overweight increased from 17.4% to 27.5%[4]. Obesity and overweight are associated with an increasing incidence of a number of conditions, including diabetes mellitus, cardiovascular disease, nonalcoholic fatty liver disease, HCC and others[3], [6]. In addition, many studies[7]–[9] have suggested that obesity may have a negative impact on surgical outcomes due to associated co-morbidities, and many studies have assessed the morbidity and mortality in obese patients but the results still remain controversial. Most such studies[7], [10]–[14] were carried out in western countries and the liver resection was performed in patients with all types of liver diseases. Few studies have focused on Chinese people, in whom the prevalence of obesity and overweight has been lower. Moreover, fewer studies have researched the effect of obesity and overweight in HCC patients, in which about 80% of the cases in China occur in cirrhosis of the liver [15].

The aim of our study was to investigate the safety of overweight and obese patients undergoing liver resection for hepatitis B virus (HBV) -related HCC in a large sample.

## Method

### Population and Study Design

Between January 2009 and March 2013, 1543 consecutive HBV-related HCC patients undergoing elective liver resection were included in our study. All subjects were diagnosed with HCC by histology and with HBV infection or a history of HBV infection. Patients underwent surgery only when their Child-Turcotte-Pugh (CTP) score was A. Records for patients' data indicating demographics, comorbid conditions, laboratory values, intraoperative parameters and postoperative outcomes were collected prospectively in the HCC of West China Hospital of Sichuan University database (HCCWCHSU System). The protocol was approved by the West China Hospital Ethical Committee and written informed consent was obtained from all recipients before inclusion. Based on body mass BMI categories of WGOC[5], [16] criteria, the patients were stratified into an underweight group (BMI<18.5), a normal weight group(BMI:18.5–23.9), an overweight group(BMI:24.0–27.9) and an obese group(BMI≥28). The primary outcomes of this study were the mortality and postoperative complications in the four groups. The secondary outcome was the co-morbidities among the four groups.

### Perioperative Management

All of the patients were managed by the same surgical team. All patients underwent a thorough history enquiry, physical examination and routine preoperative laboratory measurements. Echocardiography, chest radiograph or computed tomography, pulmonary function test and coronary angiography were carried out, if necessary. Routine preoperative imaging examinations to evaluate the tumor included contrast computed tomography or magnetic resonance imaging of the abdomen. Patients undergoing surgery were administered under general anaesthesia and were explored through an extended right subcostal incision, intraoperative ultrasonography was performed routinely. Hemihepatic vascular inflow occlusion[17] or the Pringle maneuver[18] were used according to the surgeon's preference in most patients. Liver parenchymal transection was performed using the hooking ligation technique or an ultrasonic dissector with coagulator[17]. Based on preoperative and intraoperative condition, patients were transferred to the intensive care unit for treatment if necessary.

### Definition of the Parameters

The Clavien-Dindo complications classification system[19] was used to grade postoperative complications. Complication grade III-V is defined as severe complication. Liver resection of more than three segments was defined as major resection, and liver resection of fewer than three segments was defined as minor resection[20]. Portal hypertension was defined as esophageal varices detected by endoscopy or splenomegaly (major diameter >12 cm) with a platelet count <100 000/mm^3^ according to the Barcelona Clinic Liver Cancer Group criteria[1]. For individual preexisting disease, we used the Charlson index[21], [22] to quantify comorbidities. Extrahepatic procedure included all other operations except for liver resection, such as bowel resection, adrenalectomy, diaphragm resection, biliary tract exploration and adhesion separation due to reoperation. Cardiovascular disease included coronary heart disease, previous coronary revascularization, cerebral arterial occlusive disease, and/or peripheral vascular occlusive disease. Pulmonary disease indicated chronic obstructive pulmonary disease, asthma, chronic bronchitis and tuberculosis. Wound complications included infections, hematoma and wound dehiscence. The definition and grading of severity of liver failure were based on International Study Group of Liver Surgery criteria[23]. Histologic staging of fibrosis was performed using the Ishak fibrosis score[24].We used 30-day mortality and 90-day mortality to evaluated the perioperative mortality.

### Statistical Analysis

Statistical analysis was performed using SPSS Version 17 statistical analysis software and significances waa set at P<0.05. Continuous variables were compared among groups by the Student *t-* test or Mann–Whitney U test when appropriate. The Chi-square test or Fisher's exact test were used to compare categorical variables. To identify risk factors for complications, only factors associated with complications in the univariate analysis with significant difference entered into a forward stepwise logistic regression analysis.

## Result

### Patient characteristics

Our cohort consisted of 1543 HBV-related HCC patients, with BMIs ranging from 16.4 kg/m^2^ to 35.0 kg/m^2^, and a mean of 22.9 kg/m^2^.Of the subjects, 73 (4.7%) were obese, 412 (26.7%) were overweight, 982 (63.6%) were normal weight and 76 (4.9%) were underweight according to the categories of WGOC criteria. According to the distribution of BMI, we categorized all the patients into four levels. The patient characteristics of the four groups were displayed in Table 1. The gender distribution, the proportion of Charlson index >3, comorbidity and hypertension were significantly different among the four BMI groups. Overweight patients had higher proportion of male subjects (88.8% vs 83.4%, P = 0.01) than the normal weight group and underweight patients had a lower proportion of male subjects than the normal weight group(55.3% vs 88.8%, P<0.001). Compared to patients with normal weight, overweight patients had a higher incidence of comorbidities (35.2% vs 26.3%, P = 0.001), Charlson index >3(13.6% vs 7.8%,P = 0.001) and hypertension(24.8% vs 14.7%, P<0.001). The obese patients also had a higher incidence of comorbidities(38.4% vs 26.3%, P = 0.025) and hypertension(31.5% vs 14.7%, P<0.001) compared to patients with normal weight.

**Table 1:** Preoperative Characteristics of Patients who underwent Hepatectomy.

Clinical characteristics	Underweight	Normal Weight	Overweight	Obesity	P Value
	(BMI<18.5)	(BMI:18.5–23.9)	(BMI:24.0–27.9)	(BMI≥28)	
	(n = 76)	(n = 982)	(n = 412)	(n = 73)	
Male (%)	42*(55.3%)	819(83.4%)	366*(88.8%)	67(91.8%)	<0.001
Age (year), median(IQR)	53.5(42–63)	50(41–59)	49(41.25–59)	47(40.5–56)	0.228
ASA grade ≥III (%)	8(10.5%)	143(14.6%)	64(15.5%)	13(17.8%)	0.605
Charlson index >3,(%)	10 (13.2%)	77(7.8%)	56*(13.6%)	8(11.0%)	0.007
Comorbidity (%)	17(22.4%)	258(26.3%)	145*(35.2%)	28*(38.4%)	0.001
Hypertension (%)	8(10.5%)	144(14.7%)	102*(24.8%)	23*(31.5%)	<0.001
Pulmonary disease (%)	4(5.3%)	30(3.1%)	10(2.4%)	3(4.1%)	0.600
Cardiovascular disease (%)	0(0%)	23(2.3%)	15(3.6%)	4(5.5%)	0.056
Diabetes mellitus (%)	3(3.9%)	69(7.0%)	40(9.7%)	5(6.8%)	0.205
HBsAg (%)	64(84.2%)	802(81.7%)	333(80.8%)	51(69.9%)	0.082
HBeAg (%)	12(15.8%)	145(14.8%)	76 (18.4%)	13(17.8%)	0.372
HBV DNA>2000 U/mlL(%)	22(28.9%)	301(30.7%)	137(33.3%)	23(31.5%)	0.772
Antiviral treatment before hepatectomy	15(68.2%)	211(70.1%)	101(73.7%)	17(73.9%)	0.853
AST (U/L)>ULN (%)	29(38.2%)	410(41.8%)	167(40.5%)	30(41.1%)	0.921
ALT (U/L)>ULN (%)	17(22.4%)	277(28.2%)	114(27.7%)	25(34.2%)	0.450
Hemoglobin(g/L)>120 g/L, (%)	59(77.6%)	838(85.3%)	362(87.9%)	66(90.4%)	0.070
Platelets(10^9^/L),median(IQR)	146(96–184)	130(92–185)	130(92–179)	138(90–177)	0.882
Portal hypertension (%)	21(27.6%)	273(27.8%)	112(27.2%)	22(30.1%)	0.965
Ishak score, median (IQR)median(IQR)	6(4–6)	5(4–6)	5(4–6)	5(5–6)	0.576
Tumor size(cm), median(IQR)	6(3–8)	5(4–8)	5(4–8)	6(4.5–9)	0.605
Tumor Number					0.377
Solitary	64(84.2%)	825(84%)	356(86.4%)	66(90.4%)	
Multiple	12(15.8%)	157(16%)	56(13.6%)	7(9.6%)	

^*^P<0.05 compared with the normal-weight BMI group. BMI:body mass index; HBsAg: hepatitis B surface antigen; HBeAg: hepatitis B e antigen; SD: standard deviation; ULN, upper limit of normal. ALT,alanine aminotransferase; AST, aspartate aminotransferase. ASA: American Society of Anesthesiologists category. IQR: interquartile range.

The difference in comorbidities is mainly reflected in higher incidence of hypertension. No significant differences were found for cardiovascular disease, pulmonary disease and diabetes mellitus. Other preoperative parameters such as age, American Society of Anesthesiologists category (ASA) grade, hepatitis B surface antigen(HBsAg), hepatitis B e antigen (HBeAg), HBV DNA-positive (more than 2000 U/mL), aspartate aminotransferase(AST), alanine aminotransferase (ALT),hemoglobin, platelets, Ishak score, tumor size, tumor number, rate of antiviral treatment before operation and portal hypertension were not significantly different among the four groups. All the patients with HBV DNA positive got antiviral treatment after hepatectomy, however, only 71.2%(344/483) of them had antiviral treatment before surgery.

### Intraoperative data

The four groups experienced similar operative procedures(Table 2). Anatomic resection was performed in 557 (36.1%) patients, including 223 right hemihepatectomies, 119 left hemihepatectomies,114 left lobectomies,25 mesohepatectomies and 76 other procedures. Based on intraoperative findings and pathological examination, 1430(92.7%) patients were with R0 resection, 77 (5.0%) with R1 resection and 36(2.3%) with R2 resection.The rates of major liver resection (P = 0.902) and additional extrahepatic procedures (P = 0.196) were not different among the four groups. Moreover, the methods used for liver parenchyma transection (hooking ligation or ultrasonic dissector), anatomic resection rate, packed red blood cells (PRBCs) transfusion, plasma transfusion requiremen and inflow occlusion rate during hepatectomy were also similar in the four groups. Patients with BMI≥28 seemed to have more blood loss, but there were no significant differences (P = 0.224).

**Table 2:** Intraoperative Parameters of Patients who underwent Hepatectomy by Obesity Classification.

Intraoperative Parameters	Underweight	Normal Weight	Overweight	Obesity	P value
	(BMI<18.5)	(BMI:18.5–23.9)	(BMI:24.0–27.9)	(BMI≥28)	
	(n = 76)	(n = 982)	(n = 412)	(n = 73)	
Major resection (%)	30(39.5%)	359(36.6%)	158(38.3%)	27(37.0%)	0.902
Extrahepatic procedures (%)	12(15.8%)	199(20.3%)	73(17.7%)	20(27.4%)	0.196
Hooking with ligation (%)	25(32.9%)	339(34.5%)	150(36.4%)	24(32.9%)	0.864
Inflow occlusion (%)	36(47.4%)	411(41.9%)	163(39.6%)	32(43.8%)	0.589
Blood loss (mL) media(IQR)	300(200–500)	400(200–600)	400(200–600)	430(300–525)	0.224
PRBCs transfusion (%)	16(21.1%)	177(18.0%)	83(20.1%)	12(16.4%)	0.709
Plasma transfusion (%)	11(14.5%)	136(13.8%)	70(17.0%)	11(15.1%)	0.516
Anatomic resection (%)	30(39.5%)	361(36.8%)	145(35.2%)	21(28.8%)	0.492

BMI:body mass index;SD: standard deviation.PRBCs:packed red blood cells.

### Postoperative outcomes

Postoperative complications and mortality are shown in Table 3. The overall morbidity,30-day mortality and 90-day mortality rates in our cohort were 30.1%,1.5% and 1.6%, respectively. Neither the 30-day mortality nor the 90-day mortality rate was different among the four groups. For comparison, the Clavien-Dindo complications classification system[19] was used in gradingr postoperative complications. Compared to normal weight patients, total complications were more common in overweight and obese patient, however, no significant differences were reached. The underweight patients had fewer total complication(15.8% vs 29.3%,P = 0.012).With regards to specific complications, postoperative common complications included bile leakage, liver failure, ascites, cardiovascular complication, pulmonary complications, infection complications, bleeding, wound complications and gastrointestinal complications. Differences in these complications among the four groups were not significant except for wound complications. Overweight(6.3% vs 2.5%,P<0.001) and obese patient(11.0% vs 2.5%,P = 0.001) had more wound complication than the normal weight patients. In addition, required hospital stay (days) (P = 0.382) and ICU stay (P = 0.132) were not different among the four groups. The perioperative outcomes between overweight and obese patients were similar.

**Table 3:** Postoperative Outcomes of Patients who underwent Hepatectomy.

Postoperative Outcomes	Underweight	Normal Weight	Overweight	Obesity	P value
	(BMI<18.5)	(BMI:18.5–23.9)	(BMI:24.0–27.9)	(BMI≥28)	
	(n = 76)	(n = 982)	(n = 412)	(n = 73)	
Total complications	12*(15.8%)	288(29.3%)	136(33.0%)	28(38.4%)	0.008
Major complications	3(3.9%)	82(8.4%)	31(7.5%)	9 (12.3%)	0.286
Grade I	3(3.9%)	94(9.6%)	44(10.7%)	5(6.8%)	0.261
Grade II	6(7.9%)	112(11.4%)	61(14.8%)	14(19.2%)	0.057
Grade III	0(0%)	49(5.0%)	18(4.4%)	4(5.5%)	0.054
Grade IV	1(1.3%)	22(2.2%)	6(1.5%)	2(2.7%)	0.717
30-day Mortality	2(2.6%)	11(1.1%)	7(1.7%)	3(4.1%)	0.293
90-day Mortality	2(2.6%)	12(1.2%)	8(1.9%)	3(4.1%)	0.293
Bile Leakage	0(0%)	13(1.3%)	8(1.9%)	0 (0%)	0.620
Liver failure	6(7.9%)	93(9.5%)	50(12.1%)	11(15.1%)	0.215
Grade A	4(5.3%)	32(3.3%)	11(2.7%)	1(1.4%)	0.008
Grade B	2(2.6%)	42(4.3%)	31(7.5%)	8(11%)	
Grade C	0(0%)	19(1.9%)	8(1.9%)	2(2.7%)	
Ascites	1(1.3%)	47(4.8%)	19(4.6%)	1(1.4%)	0.180
Cardiovascular complication	2(2.6%)	8(0.8%)	5(1.2%)	1(1.4%)	0.568
Pulmonary complications	3(3.9%)	61(6.2%)	29(7.0%)	6(8.2%)	0.664
Infection complications	2(2.6%)	49(5.0%)	25(6.1%)	5 (6.8%)	0.516
Bleeding	1(1.3%)	13(1.3%)	4(1.0%)	2(2.7%)	0.791
Wound complication	1(1.3%)	25(2.5%)	26*(6.3%)	8*(11.0%)	<0.001
Gastrointestinal complications	1(1.3%)	19(1.9%)	13(3.2%)	4(5.5%)	0.221
ICU stay requirement	16(21.1%)	274(27.9%)	134(32.5%)	23(31.5%)	0.132
Hospital stay(days),median (IQR)	12(10–15)	12(10–15)	12.5(10–15)	12(10–14)	0.382

P<0.05 compared with the normal-weight BMI group. BMI: body mass index; ICU: Intensive Care Unit, IQR: interquartile range.

### Prognostic factors for complications by logistic analysis

Univariate analysis showed that BMI grade was significantly correlated with postoperative complications (P = 0.008). To assess the clinical significance of BMI, we investigated prognostic factors for total complications by logistic regression. Fourteen significant variables in univariate analysis (P<0.05) were identified and they were BMI, gender, major liver resection, extrahepatic procedures, portal hypertension, PRBCs transfusion, Charlson index >3, pulmonary disease, diabetes mellitus, ASA grade ≥III, AST, ALT, blood loss and platelets. All of the 14 parameters entered into the regression model and significant variables for total complications included gender, major liver resection, extrahepatic procedures, portal hypertension, PRBCs transfusion, pulmonary disease, ASA grade ≥III, ALT and blood loss(P<0.05) (Table 4). Logistic analysis indicated that BMI was not a risk factor for total complications.

**Table 4:** Independent prognostic factors for total complications according to logistic analysis.

Variables	Odds Ratio	95% CI	P
Male	0.549	0.384–0.784	0.001
Major liver resection	1.926	1.491–2.490	<0.001
Extrahepatic procedures	1.567	1.174–2.091	0.002
Portal hypertension	2.548	1.959–3.316	<0.001
PRBCs transfusion	1.962	1.385–2.779	<0.001
Pulmonary disease	3.564	1.823–6.967	<0.001
ASA grade ≥III	2.073	1.494–2.876	<0.001
ALT (U/L)>ULN	1.318	1.019–1.704	0.036
Blood loss(mL)	1.00	1.00–1.001	0.030

ALT: alanine aminotransferase; AST: aspartate aminotransferase. PRBCs: packed red blood cells.

## Discussion

Surgeons are operating on obese patients in increasing numbers because the rate of obesity and overweight in adults has been rising all around the world in recent decades[4] and many types of cancers are related to obesity[6]. With co-morbidities and operating difficulties, obese patients seem to have worse perioperative outcomes[8]. But, the results of studies for assessing the effect of obesity on various surgeries remain inconsistent. We studied a cohort including a large sample of 1543 HCC patients to assess whether BMI has effects on postoperative outcomes. In our study, although overweight and obese patients had more co-morbidities, the two groups had similar morbidity and mortality except for wound complications compared to normal weight patients. Underweight patients and normal weight patients had similar preoperative and intraoperative parameters, but the total number of complications in underweight patients was lower. Our logistic analysis indicated that BMI was not a risk factor for complications. Moreover, our study is the first to use a large-sample cohort to investigate the safety of overweight and obese patients undergoing liver resection for HBV-related HCC in China. HCC is very common in Asia, especially in China, where 54% of all HCC occurred[15]. HCC patients usually had underlying liver damage from cirrhosis because the prevalence of cirrhosis in patients with HCC was between 80% and 90%[15], which could increase the risk of morbidity and mortality. Thus, our results are significant for liver surgeons in considering safety of liver resection on HCC patients with obesity and overweight when making clinical decisions.

It is well established that obese patients as well as overweight patients have more co-morbidities, such as cardiovascular disease and diabetes mellitus[3], [11], [12], [25]. In addition, two studies with large samples earlier indicated that obese and overweight patients did increase workload for surgeons by adding operating time or length of hospital stays[2], [26]. The findings from our study were consistent with some of the previous studies[2], [7], [9], [13], [14], not only in preoperative co-morbidities but also in postoperative outcomes. In general surgery, Dindo[9] investigated a cohort of 6336 patients and found that obese patients had similar postoperative complications except for wound infection. Similarly, Hawn et al[2] found that obesity did not increase the morbidity in a study including 1375 patients. For liver resection, Gedaly's study[26] included 1029 liver resections and showed that obese patients demonstrated an almost linear increase in risk for infectious and major noninfectious complications, but the increase did not demonstrate significance.

Although many large-sample studies evaluating the safety of obese patients have been carried out, the results are still contradictory. A prospective cohort study[25] of 118,707 patients undergoing non-bariatric general surgery showed that a progressive increase in the likelihood of a complication with increasing BMI class and patients with overweight and moderate obesity had lowest mortality. Mathur et al[27] carried out a study including 3960 liver resections and suggested that the degree of obesity is independently associated with an increasing complication rate but not with mortality. Other cohort studies focused on liver resection[7], [10]–[14], [28], [29] also drew different conclusions. There were three studies[12]–[14] exclusively investigating obesity in HCC patients. Two of these studies[13], [14] showed that the mortality and morbidity in obese and non-obese patients were similar, however, the third study[12] showed that obese patients had higher mortality.

The reasons for conflicting conclusions about the effects of obesity may be as follows. First, the main reason may be differences of inclusion criteria of patients in studies. For liver resection, the cohort with the largest sample included all types of liver diseases for hepatectomy while Cucchetti's study[14] included only patients with cirrhosis. Our cohort included patients with HBV-related HCC. The accompanying chronic liver disease in HBV-related HCC patients may cause more severe complications than in other patients[30]. Second, the absence of unified standards in reported complications hampered proper evaluation of the surgery results in several large-sample studies[2], [8], [11], [25]–[27]. Thirdly, for liver resection in HCC patients, most cohorts[12]–[14] had a small sample of patients and the statistical power was limited. We carried out a retrospective analysis of prospectively collected data on elective liver resection for HBV-related HCC patients. With a large sample of consecutive 1543 cases, our study would contribute to evaluating the risk for HCC patients with overweight or obesity.

Obesity ever was considered to have negatively impact on surgical outcomes due to associated co-morbidities[7]. In our study, the overall complications and severity classified by the Clavien system were similar between the normal and BMI>24 groups(overweight and obese patients), which did not agree with the assumed results. Several reasons can explain this unexpected outcome. First, patients with a relatively high BMI may have a higher nutritional and physiologic reserve than lower BMI patients and may thus withstand the various effects of hepatic resection coupled with other therapies [13], [28]. Second, although obese and overweight patients had more co-morbidities, the patients were selected for safety before surgery in consideration of the morbidities, ASA score and liver function. However, more wound complications occurred in the overweight and obese patients. The reasons had been discussed by Dindo[9] and Mullen[8]. First, obese patients had excessive fat tissue, which has low regional oxygen tension and relatively low blood perfusion, and may increase the chance for wound to infect. Second, obese patients have an underlying immune impairment[6]. And third, obese patients usually had larger incisions because of difficulty in exposure. Tension on the suture line might be more accentuated because of the bulky subcutaneous fat tissue[9].

However, a few potential limitations in our cohort should be considered. First, for BMI classification, we selected WGOC criteria, not the World Health Organization criteria, for BMI classification, and our results may not be generalizable to other countries or areas beyond China. Second, our results may not be generalizable to other patients beyond HCC patients. Third, patients were followed up for just a month and late complications such as incisional hernia can not be expected in our study.

In conclusion, we carried on a retrospective study with a large sample and found that overweight and obese patients with HBV-related HCC had more co-morbidities than did normal or underweight patients. However, overweight and obesity were not related with morbidity and mortality except for wound complication after liver resection. BMI is not a risk factor for complications after liver resection.
